# Circulating large extracellular vesicles carrying CA9 in the diagnosis and prognosis of clear‐cell renal cell carcinoma

**DOI:** 10.1002/ctm2.358

**Published:** 2021-03-27

**Authors:** Luisa Vergori, Maria Carmen Martinez, Pierre Bigot

**Affiliations:** ^1^ INSERM U1063 SOPAM University of Angers Angers France; ^2^ Department of Urology Angers University Hospital Angers France

Dear Editor,

The increasing use of highly sensitive whole‐body imaging technologies for solid lesions of the kidney poses no diagnostic difficulty, but, the lack of adequate methods to detect cancers at early stages exhibits outcomes of unclear clinical significance. In these instances, biomarkers could be very helpful for an accurate diagnosis.^1^ Carbonic anhydrase 9 (CA9) is up to date found to be the most promising biomarker for renal cell carcinoma (RCC), especially for clear‐cell RCC (ccRCC). CA9 is a metalloenzyme overexpressed in almost all ccRCC following hypoxia (via HIF1) and inactivation of the VHL gene, but absent in benign tumors and normal renal tissue.^2^ Immunohistochemistry of CA9 in preoperative renal biopsy is the major method routinely performed in pathology laboratory. Beyond these invasive techniques, the integration of blood‐based assays such as the serum concentration of total (t)‐CA9 by ELISA^3^ in clinical setting is still impractical.

Large extracellular vesicles (lEVs) represent a new source of noninvasive cancer biomarkers from liquid biopsies.^4^ They are secreted by almost all cells and involved in tumor progression.^5^ It has been reported that the potential of tumor‐derived lEVs into the bloodstream may be exploited for diagnosis.^6^ Here, by flow cytometer, we evaluate expression levels of CA9 in tumor‐associated lEVs in plasma from RCC patients and healthy controls.

The study included 77 RCC patients and 16 healthy controls (Figure S1). The characteristics of the individuals of the study are summarized in Table S1. Among patients without metastasis at diagnosis, 11 experienced a local or a metastatic recurrence.

First, we characterized lEVs following MISEV guidelines^7^ (Figures S2A and S2B). Next, to assess the feasibility of using lEV quantification in clinical situation, we detected CA9 as circulating lEV cargo component by flow cytometry (Figure 1A). We also compared this method whit ELISA assay (t‐CA9). The percentage of lEVs expressing CA9 (CA9^+^‐lEVs) and t‐CA9 levels (Figures 1A and 1B) was significantly higher in plasma from RCC patients compared to those from controls. CA9^+^‐lEVs as well as the plasma t‐CA9 levels in ccRCC patients were significantly higher than that in healthy controls (Figures 1C and 1D). Interestingly, changes of expression levels of CA9^+^‐lEVs in ccRCC patients occurred 1 month after surgical removal of the tumors, indeed the signal of CA9 obtained by flow cytometry was reduced postoperatively (Figure S3), suggesting that the circulating CA9^+^‐lEVs could be released from the primary renal tumors into plasma. Moreover, in ccRCC CA9^+^‐lEVs correlated with tumor dimension (Figure 1E) and with ISUP grade I–II versus III–IV (Table S2).

**FIGURE 1:**
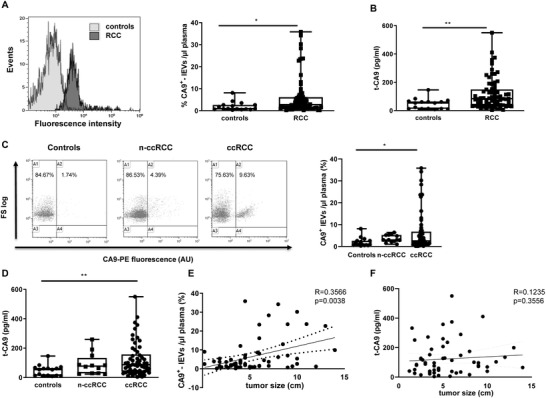
Flow cytometric detection of circulating lEVs by staining for the tumor‐associated cell surface markers (CA9) and concentration of t‐CA9 in RCC and ccRCC measured by ELISA. (A) Representative flow cytometer plots for CA9‐lEVs from controls and RCC. RCC exhibited a strong positive staining by antibody anti‐CA9, whereas the corresponding control sample showed a weak fluorescence signal. Mean of the percentage of CA9^+^ lEVs. (B–D) Plasma t‐CA9 concentration expressed in pg/ml in RCC, renal cell carcinoma, and clear‐cell renal cell carcinoma (ccRCC). (C) Summarized data on the percentage of CA9^+^ lEVs in RCC subgroups, n‐ccRCC and ccRCC. The percentages show the number of positive events for staining of plasma circulating lEVs visualized by plotting CA9 marker (*x*‐axis) versus FS log properties (*y*‐axis) and gated based on isotype control. (E–F) Spearman correlations were performed between percentage of lEVs expressing CA9 detected by flow cytometry, plasma concentration of t‐CA9 observed by ELISA, and tumor size (cm). Data are shown as mean values ± SEM. *P < 0.05 and **P < 0.01

Conversely, the plasma t‐CA9 levels detected by ELISA did not correlate with the tumor dimension measured at pathologic examination in ccRCC patients (Figure 1F), with the ISUP grade or the pathologic stage (Table S3). In accordance with Liao et al.,^8^ plasma circulating CA9^+^‐lEVs, but not plasma t‐CA9 levels, correlated with histological subtype of RCC, tumor size, and grade.

Besides, a predicative value of quantitative CA9^+^‐lEV levels (>1.85%) in ccRCC was assessed using an ROC curve (Table S4) and compared with ELISA assay. The area under ROC of circulating CA9^+^‐lEVs was 0.70 (95% CI, 0.57‐0.84) and a sensitivity of 68.8% and specificity of 60.9% (Figure S4A). Based on ROC analysis, 88.6% positive predictive value of individuals who achieve a higher cutoff of 1.85% on the flow cytometry was accurately diagnosed with ccRCC. Conversely, 30.7% negative predictive value of individuals who achieve a cutoff of 1.85% or lower was accurately diagnosed as healthy. This threshold value of 1.85% produced a strong efficiency for predicting outcomes, with Yule's *Q* coefficients of 0.55 and a *χ*2 test ≤ 0.05 (Table S4). In Figure S4B and Table S4, the results using t‐CA9 measured by ELISA at threshold value of 60.8 pg/ml showed similar flow cytometry performance. Despite these similar accuracy performances, flow cytometry sensitivity was better than ELISA. Therefore, we believe that application of level of CA9‐lEVs measured by flow cytometry in the plasma for cancer diagnosis is promising.

Moreover, a numeration in absolute value of circulating CA9^+^‐lEVs was also performed by flow cytometry on patients. The correlation between clinical and pathological characteristics, the number of CA9^+^‐lEVs detected by flow cytometry, and t‐CA9 concentration measured by ELISA are reported in Table 1. To further evaluate the prognostic value of CA9^+^‐lEVs, RCC patients were divided into high‐ and low‐CA9 groups according to the median value of the numbers of CA9^+^‐lEVs. The median of the numbers of CA9^+^‐lEVs determined by flow cytometry was 350 (33–47,328) and the median value of t‐CA9 concentration quantified by ELISA was 88 (4–550) pg/ml. Tumor size (*P* = 0.05) and tumor recurrence (*P* = 0.006) were correlated with high value of CA9^+^‐lEVs.

**TABLE 1:** RCC patient and tumor characteristics according to median levels of lEVs carrying CA9 and t‐CA9 measured by flow cytometry and ELISA, respectively

	*n* = 76			*n* = 70		
	CA9‐lEVs < 350	CA9‐lEVs ≥ 350	*P*	t‐CA9 < 88	t‐CA9 ≥ 88	*P*
Gender			1			1
Female	13	11		11	10	
Male	27	25		26	23	
Median age (Year, SD)	62.8 (12)	63.8 (11)	0.717	62 (13)	65 (11)	0.275
T Stage			0.295			0.455
1	22	14		19	16	
2	1	4		4	1	
3	15	15		11	15	
4	1	2		1	1	
Histological subtype			1			0.23
Clear renal cell carcinoma	33	30		28	29	
Others	7	6		37	33	
ISUP grade			0.834			0.681
1	3	2		2	3	
2	19	14		17	16	
3	10	11		10	6	
4	6	7		5	7	
Metastasis			0.117			1
0	35	35		33	32	
1	4	0		2	1	
Median tumor size (cm, SD)	4.95 (2.5)	6.26 (3.3)	0.05	5.42 (2.9)	5.42 (3)	0.99
Recurrence*	1	11	0.006	3	7	0.139

Note: The median was used to divide the patients into high‐ and low‐CA9 groups.

Abbreviation: SD, standard deviation.

* For patient without metastasis at diagnosis.

Kaplan–Meier analysis revealed that RCC patients with high number of CA9^+^‐lEVs (>350 CA9^+^‐lEVs/μl plasma) had a worst progression‐free survival (PFS) (*P* = 0.01) compared to patients with low CA9^+^‐lEV number (<350 CA9‐MVs/μl plasma) (log rank test, *P* < 0.01) (Figure 2A), whereas high concentration of t‐CA9 (>88 pg/ml) measured by ELISA did not reach significance (*P* = 0.089) (Figure 2B). In agreement with the study of Papworth et al.,^9^ we showed higher plasma t‐CA9 concentration in ccRCC compared to other RCC types, but we did not find a significant prognostic association between plasma t‐CA9 and the PFS of ccRCC patients. Therefore, although the prognostic value of CA9 in RCC is still in debate,^10^ these results suggested that the detection of CA9^+^‐lEVs in the peripheral blood might be a potential prognostic marker in RCC.

**FIGURE 2:**
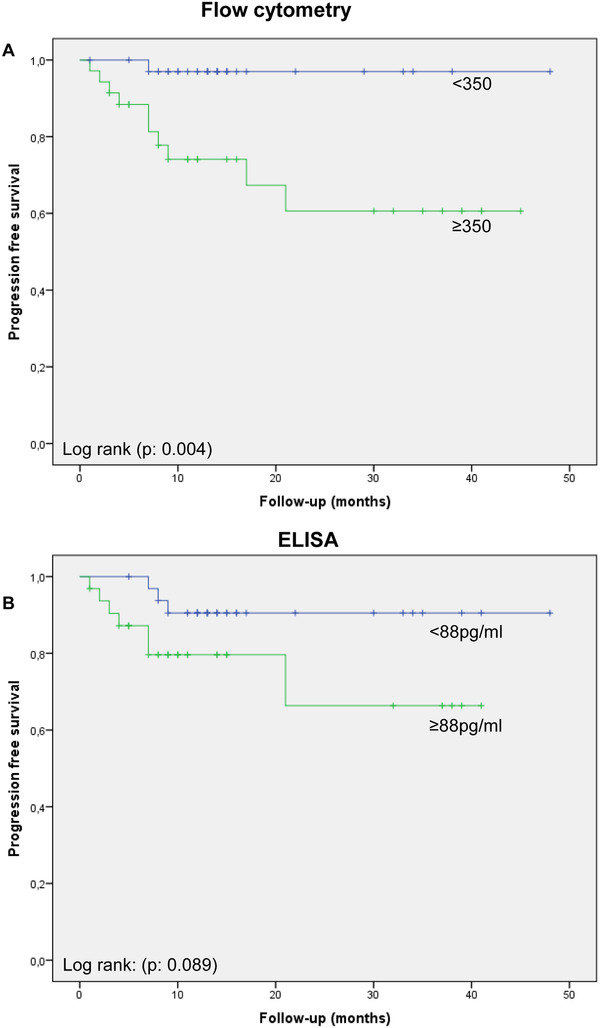
Progression‐free survival of all patients according to the number of circulating CA9^+^‐lEVs and t‐CA9 concentration detected by ELISA. (A) Localized RCC patients with low number of CA9^+^‐lEVs (blue line) revealed a better progression‐free survival than those with high value (green line). (B) No correlation was observed between t‐CA9 concentration measured by ELISA and the progression‐free survival in these patients

In conclusion, we propose that flow cytometry is a fast, reliable, quantitative, and low‐cost analysis for detection of CA9 carried by circulating renal tumor‐derived lEVs. Besides, this method displays other relevant advantages, including lower risk of complication associated to its minimal invasiveness, reproducibility, and accuracy. Interestingly, this method can be undertaken with reduced sample volume and with low consumption of reagent. Based on these results, the detection of CA9^+^‐lEVs by flow cytometry may represent an attractive and clinically beneficial test for cancer screening in the early stage, subtype classification, and in the search of risk of recurrences. Larger scale studies could permit to improve the sensitivity, specificity, and applicability of this detection method in the future.

## ETHICS APPROVAL AND CONSENT TO PARTICIPATE

Our study is approved by Biomedical Ethics Committee at Angers Hospital, CNIL authorization number DR‐2013‐206. Written consent forms have been obtained from all participants.

## AUTHOR CONTRIBUTIONS

M. Carmen Martinez and Pierre Bigot conceived and designed the study. Luisa Vergori performed the experiments and analyzed the data and prepared the figures. Luisa Vergori, M. Carmen Martinez, and Pierre Bigot interpreted their results. Luisa Vergori drafted the manuscript. Luisa Vergori, M. Carmen Martinez, and Pierre Bigot edited and approved the final version of the manuscript.

## CONFLICT OF INTEREST

The authors declare no conflict of interest.

## DATA AVAILABILITY STATEMENT

The data and other items supporting the results in the paper will be made available upon reasonable request to the corresponding authors.

## Supporting information

Supporting information

Supporting information

Supporting information

Supporting information

Supporting information

Supporting information

## References

[1] Tarnoki DL , Tarnoki AD , Richter A , Karlinger K , Berczi V , Pickuth D . Clinical value of whole‐body magnetic resonance imaging in health screening of general adult population. Radiol Oncol. 2015;49:10‐16. 25810696 10.2478/raon-2014-0031 PMC4362601

[2] Tostain J , Li G , Gentil‐Perret A , et al. Carbonic anhydrase 9 in clear cell renal cell carcinoma: a marker for diagnosis, prognosis and treatment. Eur J Cancer. 2010;46:3141‐3148. 20709527 10.1016/j.ejca.2010.07.020

[3] Takacova M , Bartosova M , Skvarkova L , et al. Carbonic anhydrase IX is a clinically significant tissue and serum biomarker associated with renal cell carcinoma. Oncol Lett. 2013;5:191‐197. 23255918 10.3892/ol.2012.1001 PMC3525455

[4] Zeuschner P , Linxweiler J , Junker K . Non‐coding RNAs as biomarkers in liquid biopsies with a special emphasis on extracellular vesicles in urological malignancies. Expert Rev Mol Diagn. 2020;20:151‐167. 31499007 10.1080/14737159.2019.1665998

[5] Kaid C , Assoni A , Marçola M , et al. Proteome and miRNome profiling of microvesicles derived from medulloblastoma cell lines with stem‐like properties reveals biomarkers of poor prognosis. Brain Res. 2020;1:1730‐146646. 10.1016/j.brainres.2020.146646 31917138

[6] Giusti I , D'Ascenzo S , Dolo V . Microvesicles as potential ovarian cancer biomarkers. Biomed Res Int. 2013;2013:703048. 23484144 10.1155/2013/703048 PMC3581088

[7] Théry C , Witwer KW , Aikawa E . Minimal information for studies of extracellular vesicles 2018 (MISEV2018): a position statement of the International Society for Extracellular Vesicles and update of the MISEV2014 guidelines. J Extracell Vesicles. 2018;7:1535750. 30637094 10.1080/20013078.2018.1535750 PMC6322352

[8] Liao SY , Aurelio ON , Jan K , Zavada J , et al. Identification of the MN/CA9 protein as a reliable diagnostic biomarker of clear cell carcinoma of the kidney. Cancer Res. 1997;57:2827‐2833. 9230182

[9] Papworth K , Sandlund J , Grankvist K , et al. Soluble carbonic anhydrase IX is not an independent prognostic factor in human renal cell carcinoma. Anticancer Res. 2010;30:2953‐2957. 20683038

[10] Zhang BY , Thompson RH , Lohse CM , et al. Carbonic anhydrase IX (CAIX) is not an independent predictor of outcome in patients with clear cell renal cell carcinoma (ccRCC) after long‐term follow‐up. BJU Int. 2013;111:1046‐1053. 23551810 10.1111/bju.12075

