# Supplementary figures and images for: Circulating large extracellular vesicles carrying CA9 in the diagnosis and prognosis of clear‐cell renal cell carcinoma
Source: Clin Transl Med. 2021 Mar 27;11(3):e358. doi: 10.1002/ctm2.358 (PMC8002908; doi:10.1002/ctm2.358)

**Figure S1**

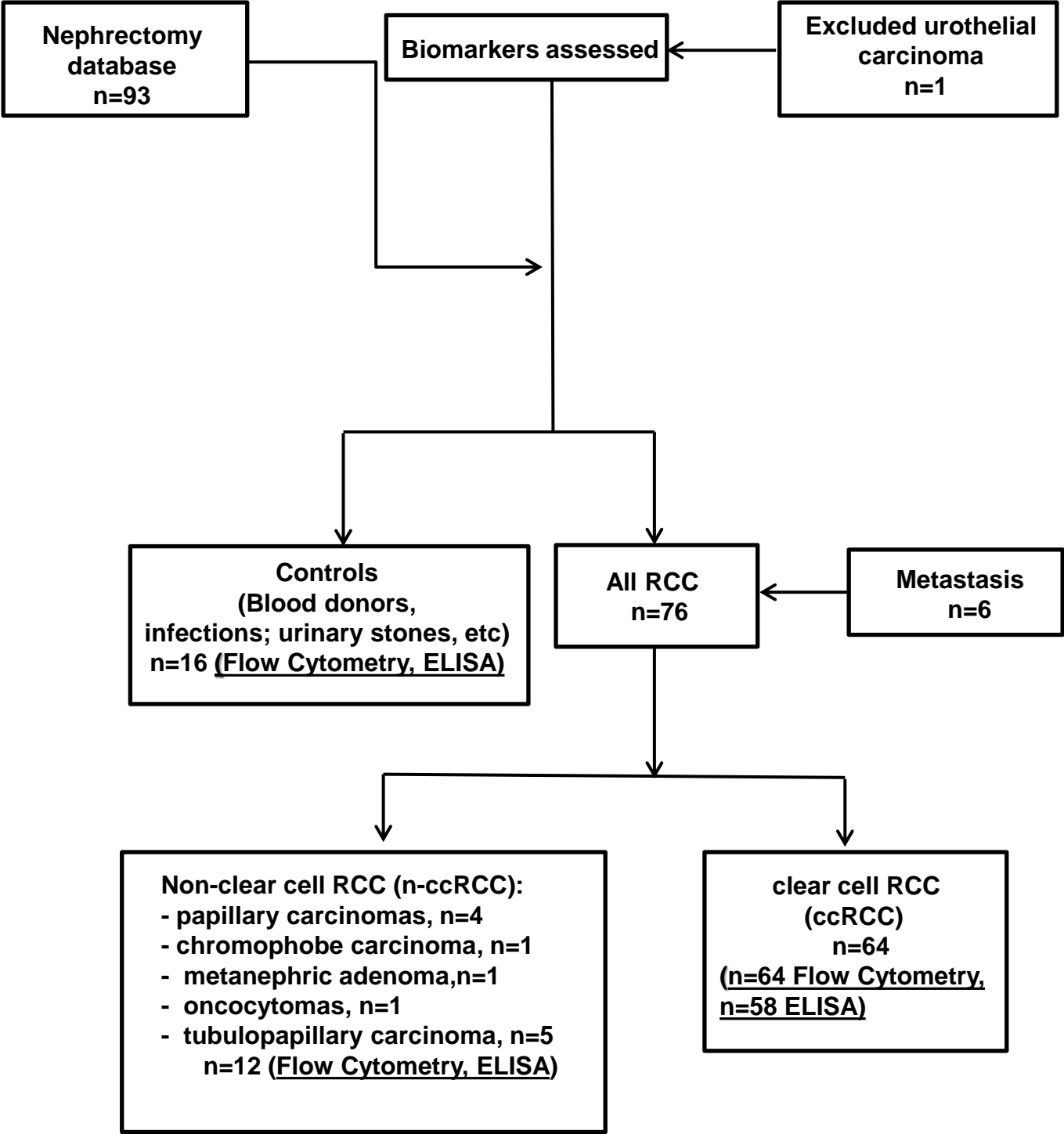

Supplement: Supplementary file 1 — Supporting information [file CTM2-11-e358-s002.pdf]

Figure S2

A

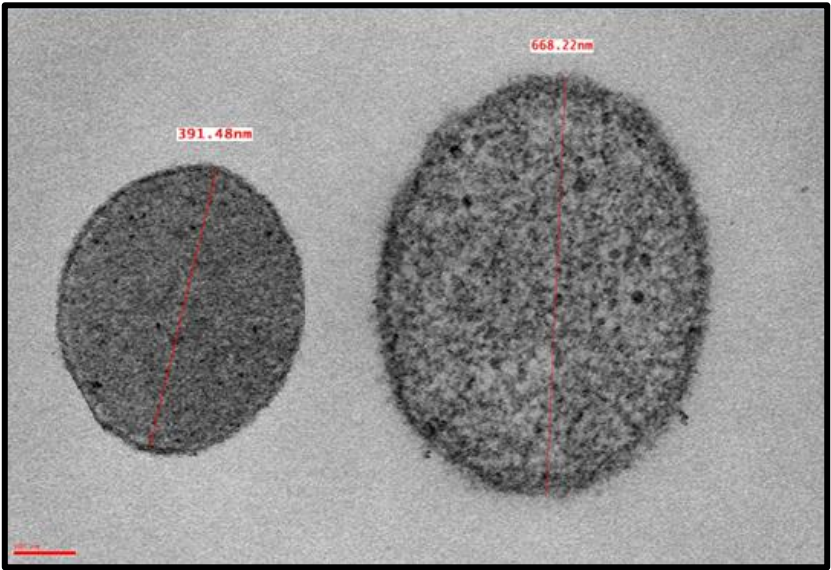

B

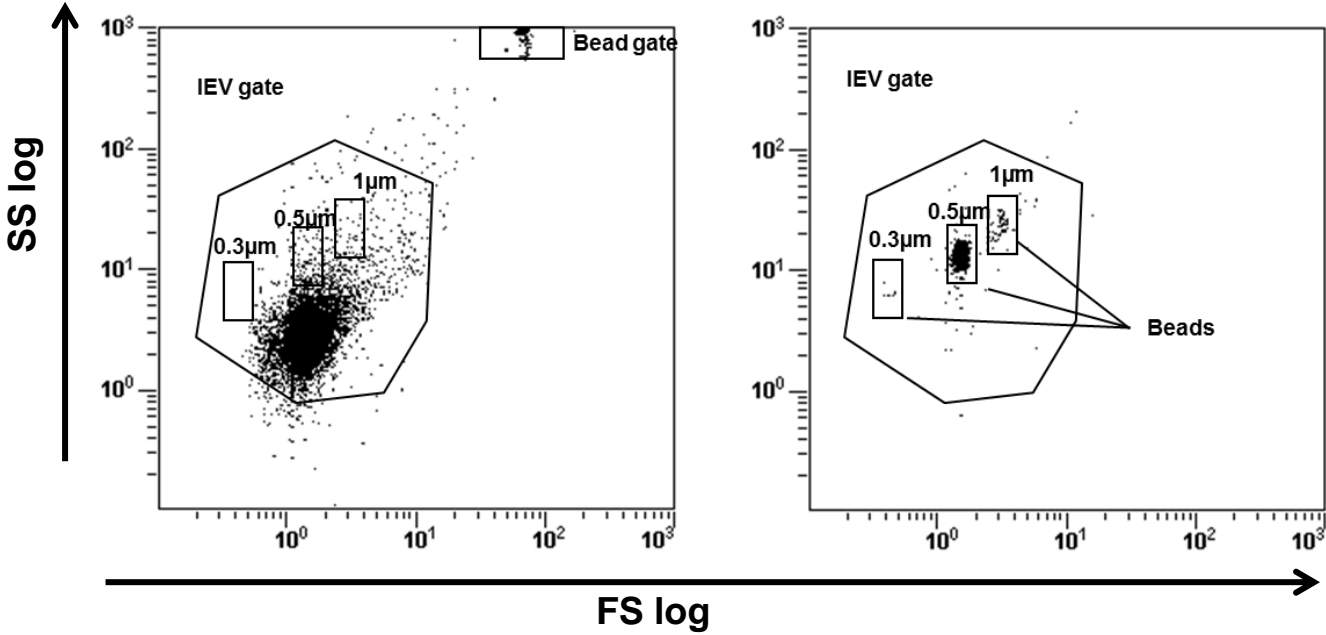

Supplement: Supplementary file 2 — Supporting information [file CTM2-11-e358-s001.pdf]

Figure S3

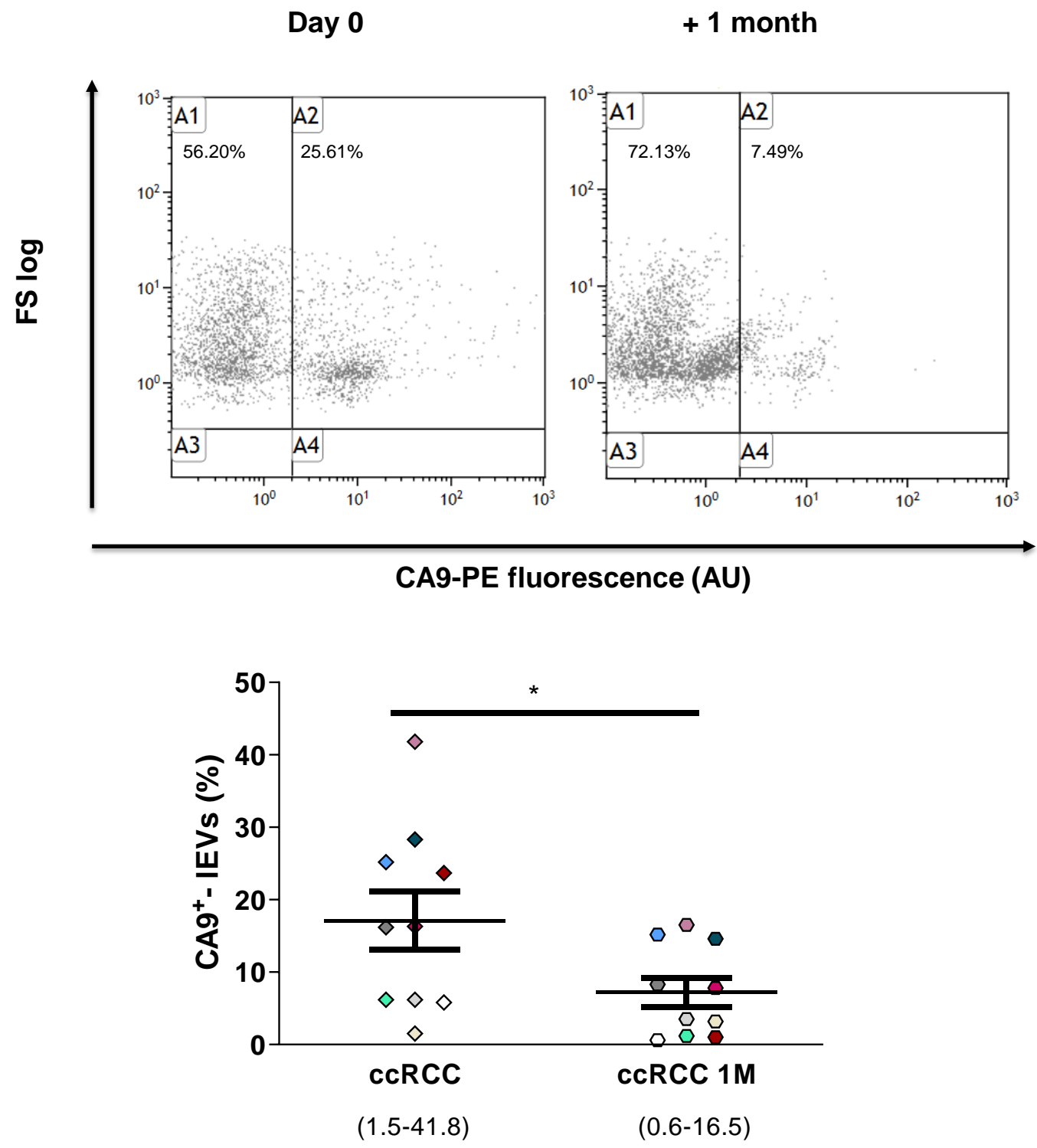

Supplement: Supplementary file 3 — Supporting information [file CTM2-11-e358-s003.pdf]

Figure S4

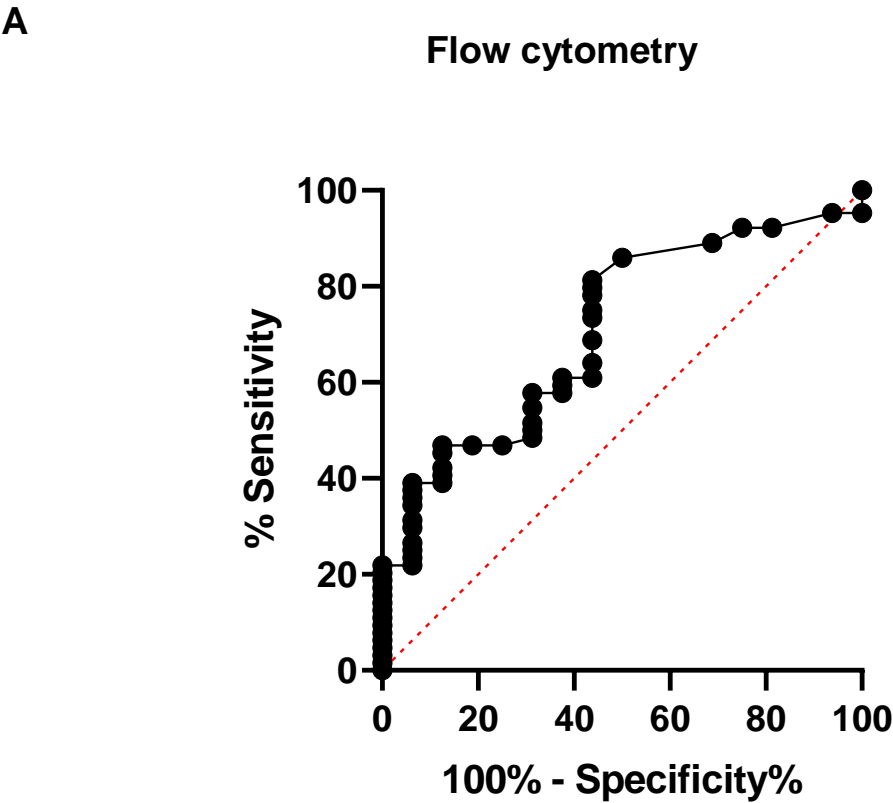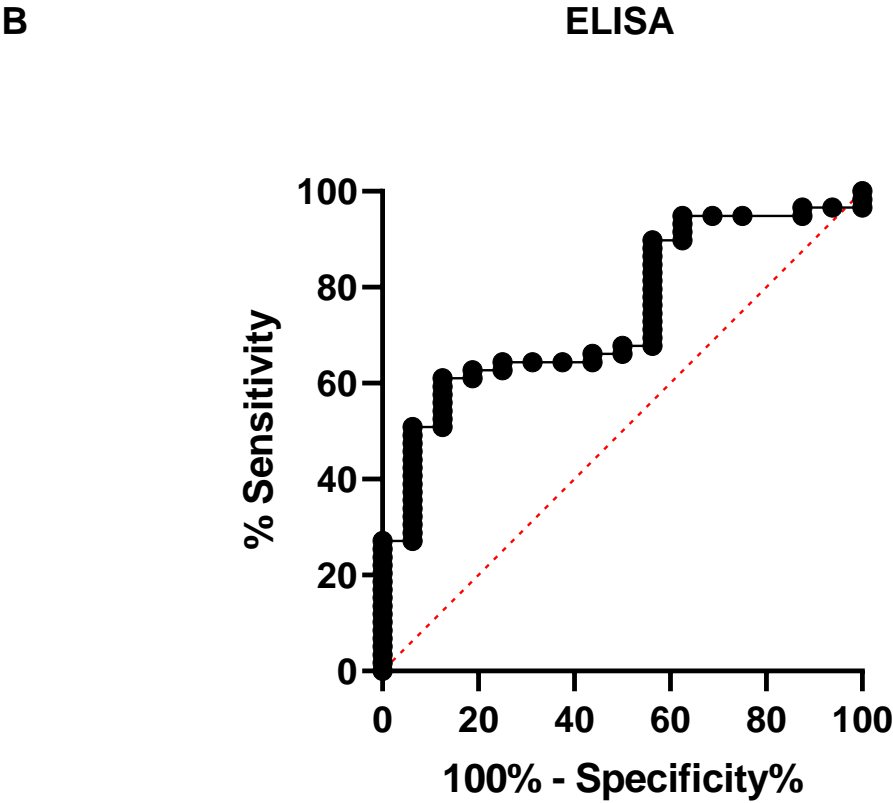

Supplement: Supplementary file 4 — Supporting information [file CTM2-11-e358-s006.pdf]
