# Supplementary material for: Circulating large extracellular vesicles carrying CA9 in the diagnosis and prognosis of clear‐cell renal cell carcinoma
Source: Clin Transl Med. 2021 Mar 27;11(3):e358. doi: 10.1002/ctm2.358 (PMC8002908; doi:10.1002/ctm2.358)
Supplement: Supplementary file 5 — Supporting information [file CTM2-11-e358-s005.docx]

**Table S1. Patient and tumor characteristics.**

| **Characteristics** |  | **controls** | **n-ccRCC** | **ccRCC** |
| --- | --- | --- | --- | --- |
| **Age at diagnosis (years)** | Median (IQR) | 40  range (24-66) | 72  range (52-80) | 65  range (38-85) |
| **Gender** | Male | 10 | 9 | 42 |
|  | Female | 6 | 3 | 22 |
| **TNM stage** | T1-T2 | - | 10 | 32 |
|  | T3-T4 | - | 1 | 32 |
| **ISUP grade** | I-II | - | 6 | 32 |
|  | III-IV | - | 2 | 32 |
| **Size** | ≤ 4 cm | - | 4 | 27 |
|  | > 4 cm | - | 8 | 37 |
| **Recurrence** |  |  | 2 | 13 |
| **Cancer-related death** |  |  | 1 | 4 |

n-ccRCC, other types of renal cell carcinoma; ccRCC, clear cell renal cell carcinoma. IQR, interquartile range; TNM, (T) tumor, (N) node, (M) metastasis; ISUP, International Society of Urological Pathology

**Table S2. Relationship between clinical characteristics and levels of circulating lEVs carrying CA9.**

| **Parameter** | **ccRCC Patients (n)** | **CA9^+^ lEVs** | |
| --- | --- | --- | --- |
|  |  | **Mean** | **P-value** |
| **Gender:** |  |  |  |
| **male** | 42 | 7.3 | 0.36 |
| **female** | 22 | 4.09 |  |
| **Age:** |  |  | 0.72 |
| **≥ 60** | 39 | 6.13 |  |
| **< 60** | 25 | 6.93 |  |
| **TNM Stage:** |  |  | 0.65 |
| **T1-T2** | 32 | 5.11 |  |
| **T3-T4** | 32 | 7.78 |  |
| **ISUP grade:** |  |  | 0.05 |
| **I-II** | 32 | 4.3 |  |
| **III-IV** | 32 | 8.56 |  |

ccRCC, clear cell renal cell carcinoma; TNM, (T) tumor, (N) node, (M) metastasis; ISUP, International Society of Urological Pathology

**Table S3. Relationship between t-CA9 levels measured by ELISA in plasma and clinico-pathological variables.**

| Plasma t-CA9 | | | | | | |
| --- | --- | --- | --- | --- | --- | --- |
|  | **ccRCC TNM stageT1-T2** | **ccRCC TNM stage T3-T4** | **P-value** | **ccRCC ISUP grade I-II** | **ccRCC ISUP grade II-III** | **P-value** |
| Case number | 31 | 27 |  | 32 | 25 |  |
| Mean (pg/mL) | 112 | 135.6 | 0.47 | 140.2 | 103.9 | 0.41 |
| Range (pg/mL) | 3.82-357.5 | 16.18-550.3 |  | 3.82-550.3 | 5-410.8 |  |

ccRCC, clear cell renal cell carcinoma. TNM, (T) tumor, (N) node, (M) metastasis; ISUP, International Society of Urological Pathology

**Table S4. Test characteristics of CA9^+^ lEVs detected by flow cytometry and t-CA9 measured by ELISA for prediction of ccRCC**

|  | **tp** | | **tn** | **fp** | **fn** | **Sensitivity (95% CI)** | **Specificity (95% CI)** | **PPV(%)** | **NPV(%)** | **Yule’s Q coefficient** | **Youden’s index** | **Chi^2^ test** | **Accurancy%** |
| --- | --- | --- | --- | --- | --- | --- | --- | --- | --- | --- | --- | --- | --- |
| CA9^+^ MVs  (flow cytometry)  t-CA9 (ELISA) | | 39  42 | 11  10 | 5  6 | 25  22 | 68.8  (41.3-88.9)  64.4  (52.7-77) | 60.9  (47.9-72.9)  62.5  (35.4-84.8) | 88.6  87.5 | 30.7  31.3 | 0.55  0.52 | 0.3  0.3 | ≤0.05  ≤0.05 | 62.5  65 |

tp =true positives; tn =true negatives; fp=false positives; fn = false negatives; PPV=positive predictive value; NPV=negative predictive value

**Table S5: Configuration of flow cytometer and settings relevant to MVs analysis**

| **Parameter** | **Value** |
| --- | --- |
| **Threshold FSC** | The value of the FSC threshold level was zero. Under these conditions, less of 6 events per second in filtered NaCl were detected |
| **PMT filter sets** | 561/10 FS detector/ 561/10 SS detector |
| **Voltage** | FSC: 1000/ SSC: 1000 |
|  | FL2 (561 nm laser): 457 |
| **Gain** | FSC: 1/ SSC: 5 |
|  | FL2: 1 |
| **Laser Power** | 6.73mW |
