# Supplementary material for: Circulating large extracellular vesicles carrying CA9 in the diagnosis and prognosis of clear‐cell renal cell carcinoma
Source: Clin Transl Med. 2021 Mar 27;11(3):e358. doi: 10.1002/ctm2.358 (PMC8002908; doi:10.1002/ctm2.358)
Supplement: Supplementary file 6 — Supporting information [file CTM2-11-e358-s004.docx]

**Supplemental Method**

**Samples**

The clinical data reported for the study were collected within the framework of the UroCCR project (NCT03293563), CNIL authorization number DR-2013-206. This prospective monocentric study included all patients treated surgically for a localized renal tumor between May 2017-January 2019 and for whom a blood sample was collected before tumor resection.

Tumors were classified according to the TNM 2009 classification^1^, histological subtypes were recorded according to the 2015 WHO classification of kidney tumors^2^ ISUP grade for RCC^3^ was analyzed. The date of nephrectomy was considered as the start of follow-up.

In total, 16 individuals served as controls; 8 of these had been admitted to the hospital for other urological conditions than RCC (infections, urinary stones, etc.) and 8 were obtained from healthy blood donors.

Peripheral blood (8ml) was collected in EDTA-treated tubes (Vacutainers, Becton Dickinson, Le Pont de Claix, France) from a peripheral vein using a 21-gauge needle to minimize platelet activation and were processed for assay within 2hours^4^. Blood was centrifuged at 260*g* for 15minutes and plasma was separated from whole blood. Then, plasma was collected and further centrifuged at 1500*g* for 20minutes to obtain platelet-poor plasma (PPP) and stored at -80°C until subsequent use. Remaining PPP was centrifuged at 17,000*g* for 45 minutes to isolate lEVs. Then, lEVs were washed in NaCl and centrifuged at 17,000*g* for 45 minutes. lEVs were resuspended in 200 μl of NaCl and stored at 4°C.

lEVs were characterized following the MISEV guidelines^5^ using transmission electronic microscopy and flow cytometry.

**Transmission electronic microscopy**

lEVs preparations were first fixed overnight at 4°C with 2.5% glutaraldehyde (LFG Distribution, Lyon, France) in 0.1 M PBS. Then, lEVs were washed two times in NaCl by 17,000*g* centrifugation for 45 minutes and dehydrated in graded alcohols. The pellet of lEVs was then embedded in Epon resin. Sections were cut and observed with a Jeol JEM 1400 microscope (Jeol, Croissy sur Seine, France) operated at 120 keV.

**Characterization of lEVs harboring CA9**

Characterization of plasma lEVs was performed by flow cytometry (500 MPL system, Beckman Coulter, Villepinte, France) using specific antibody against CA9-PE, (Cat#130-110-057, Miltenyi Biotec, Bergisch Gladbach, Germany). PPP (8 μL) was incubated with antibody (5µL) for 30 minutes in the dark at 4°C (in concentrations found to be optimal in preliminary experiments) or an equal concentration of isotype control (IgG_1_ Antibody, anti-human, PE, Cat#130-119-964, Miltenyi Biotec, Bergisch Gladbach, Germany), always done in parallel. After incubation, 200µL of NaCl was added to the tube.

The optimal configuration and settings for quantitative and qualitative flow cytometry analyses of lEVs have been adapted by previously study of Nolte-'T Hoen et al^6^ and listed in [Table S5.](https://www-sciencedirect-com.proxy.insermbiblio.inist.fr/science/article/pii/S1549963411003637?via%3Dihub#ec0010) Forward (FSC) and side (SSC) scatter of light was set in log scale, and threshold was set at the FS parameter. lEVs gating was established using varying sizes of standard microbeads, (0.1-1 μm; Megamix, Biocytex). The lEV gate included 0.3-1.0-μm beads (Figure S2B). Events in the lEV gate were further assessed for labeling with antibody against CA9-PE to distinguish true events from electronic noise and thereby increase the specificity of lEV detection.

lEV concentration was calculated using calibrated 10µm-sized Flowcount beads (Beckman Coulter) as external standard in the measurement. The population of these beads could be detected as shown in the bead gate Figure S2 B. The number of CA9^+^-lEVs was calculated after correction for the number of lEVs staining with the IgG_1_ isotype (negative control).

**Quantitative analysis of plasma CA9 levels**

Plasma CA9 was quantified by Human carbonic anhydrase 9 Quantikin ELISA kit (Cat# DCA900, R&D Systems, Minneapolis, MN, USA) according to the protocol of the manufacturer. Total CA9 corresponds to circulating levels of protein analyzed in platelet-poor plasma (PPP) fraction containing not only the potential EV-free CA9 form but also CA9 associated to lEVs and exosomes

**Statistical analysis**

Non-parametric methods (Kruskal-Wallis test and Mann-Whitney U-tests), Wilcoxon test, independent-sample t-tests and Chi^2^ tests were respectively used for comparisons of means and proportions between CA9 expression and clinical and pathologic parameters. Correlations were performed by Spearman correlation test. Differences were considered significant when *P*<0.05. To evaluate the diagnostic performance of CA9^+^-lEVs between ccRCC and healthy controls, receiver operating characteristic (ROC) curves were plotted and other diagnostic characteristics such as sensitivity, specificity, positive predictive value (PPV), negative predictive value (NPV), Yule’s Q coefficient, Youden’s index and the Chi^2^ test of significant variables were calculated.

Progression-free survival (PFS) was estimated using the Kaplan-Meier method for patients without metastasis and comparison was performed by the log-rank test. All analyses were performed using GraphPad prism 8.0 and SPSS version 15.0 software.

**References**

[1] Sobin LH, Gospodarowicz MK, Wittekind C. TNM classification of malignant tumours. *International Union against Cancer, éditeurs. 7th ed. Chichester, West Sussex, UK ; Hoboken, NJ: Wiley-Blackwell.* 2010. 309 p.

[2] Moch H, Cubilla AL, Humphrey PA, et al. The 2016 WHO Classification of Tumours of the Urinary System and Male Genital Organs-Part A: Renal, Penile, and Testicular Tumours. *Eur Urol.* 2016; 70: 93‑105.

[3] Delahunt B, Eble JN, Egevad L, et al. Grading of renal cell carcinoma. *Histopathology.* 2019; 74: 4-17.

[4] Agouni A, Lagrue-Lak-Hal AH, Ducluzeau PH, et al. Endothelial dysfunction caused by circulating microparticles from patients with metabolic syndrome. *Am J Pathol*. 2008. 173: 1210–1219.

[5] Théry C, Witwer KW, Aikawa E, Minimal information for studies of extracellular vesicles 2018 (MISEV2018): a position statement of the International Society for Extracellular Vesicles and update of the MISEV2014 guidelines. J Extracell Vesicles 2018 . 7:1535750.

[6] Nolte-'T Hoen EN, Van Der Vlist EJ, Aalberts M, et al*.*Quantitative and qualitative flow cytometric analysis of nano-sized cell-derived membrane vesicles *Nanomedicine*. 2012; 8: 712-720.
